# Students’ Performance in Online Learning Environment: The Role of Task Technology Fit and Actual Usage of System During COVID-19

**DOI:** 10.3389/fpsyg.2021.759227

**Published:** 2021-11-04

**Authors:** Sameera Butt, Asif Mahmood, Saima Saleem, Tayyiba Rashid, Amir Ikram

**Affiliations:** ^1^Institute of Quality and Technology Management, University of the Punjab, Lahore, Pakistan; ^2^Department of Business Studies, Namal Institute, Mianwali, Pakistan; ^3^Department of Industrial Engineering and Management, University of the Punjab, Lahore, Pakistan; ^4^Institute of Business and Management, University of Engineering and Technology, Lahore, Pakistan

**Keywords:** e-learning, overall quality, COVID-19 pandemic, task technology fit, students behavior, performance

## Abstract

The 2019 Pandemic has forced students to take online classes, increasing their stress levels and negatively impacting their academic performance. This issue urges the development of a mechanism to make online learning more effective in this nerve-racking time. Therefore, the present study has integrated the task technology fit (TTF) model and the DeLone and McLean Model of Information Systems Success (DMISM) to address the stated issue. The data were collected from 330 and 326 students of top-ranked public and private universities of Punjab, respectively. The theoretical framework was analyzed with the help of structural equation modeling (SEM) using Analysis of Moment Structures (AMOS). The findings indicate that overall quality positively predicts performance through the mediating role of user satisfaction and TTF. The overall quality also positively elevates performance through the mediating role of user satisfaction and actual usage of the system. Moreover, perceived usefulness proved to be a moderator between overall quality and user satisfaction. Finally, the expected practical and theoretical implications have also been discussed.

## Introduction

The WHO earlier in 2020 declared SARS-CoV2 viral infection as a pandemic, and it has become the greatest public health challenge globally. The social distance policy has been implemented worldwide to control it, leading to the closure of educational institutions in most countries. Educational institutions were directed to make significant and timely amendments in their present educational systems to provide education and maintain the academic progression of students. The WHO also encouraged this narrative to support remote learning where the situation is worsening in these pandemic days. As a result, the educational institutions switched the mode of education from on-campus to online learning. Even though many students admired this transition, a large number of the student body did not want to study online due to the increased workload, time management issues, and lack of technical skills. Moreover, recent research indicates that students feel stressed because of online learning and prefer face-to-face learning (Patricia, 2020; Fawaz and Samaha, 2021). Hence, the academic performance of students has been affected negatively, leading to a decline in their grades. So, it urges to understand how the satisfaction level and performance of the students can be enhanced using online learning platforms. Moreover, due to an abrupt transition from conventional classroom-based face-to-face learning to virtual learning, i.e., online learning, institutions, students, and instructors observe certain challenges. The most significant challenges encountered are delivering quality education, implementing quality systems required for online learning, and adapting the latest technologies (Almaiah et al., 2020).

Before determining the factors that can enhance the satisfaction level and performance of students in online learning, we first need to understand the basic concept of e-learning and its different components. Many words in literature have been used interchangeably for online learning like “blended learning,” “e-learning,” and “distance learning.” It has been defined as a method of providing education through the internet while using smartphones, laptops, desktops, tablets, etc. (Clark and Mayer, 2016). The governments of many countries are exerting their efforts to promote technology in education processes (Tenório et al., 2016) due to its benefits. Particularly, it saves time, enables interactive communication, increases learning effectiveness, provides up-to-date learning, delivers accurate knowledge, saves cost, facilitates flexible place option, and reduces spatial and temporal problems linked with physical learning (Aldholay et al., 2018c; Panigrahi et al., 2018; Aldholay et al., 2019). It is obvious from these advantages that online learning is productive and beneficial for the health of students, teachers, and related staff during the COVID-19 pandemic.

Thus, different researchers have put their efforts into formulating various theoretical concepts and establishing different models in the information system domain to anticipate and describe user behavior with the technology. The significant models deployed in this context are: Technology Acceptance Model (TAM) (Davis, 1989), Theory of Reasoned Action (TRA) (Ajzen and Fishbein, 1980), Theory of Planned Behavior (TPB) (Ajzen, 1985), Task Technology Fit model (TTF) (Goodhue and Thompson, 1995), DeLone and McLean Model of Information Systems Success (DMISM) (DeLone and McLean, 1992; Delone and McLean, 2003), and Unified Theory of Acceptance and Use of Technology Model (UTAUT) (Venkatesh et al., 2012). However, these models and their relevant theoretical concepts have immensely overlooked the determination of usage of information technology (Islam, 2013) with the exemption of DMISM, which estimates IT utilization by analyzing the impact of overall quality (system, information, and service quality) on user satisfaction and actual usage. Consequently, it affects performance and is used extensively to determine the effectiveness of information systems (Montesdioca and Maçada, 2014). Thus, the variables from these models were extracted in most research-based online learning to evaluate online learning and its various developed frameworks. For example, Gao et al. (2020) studied the impact of e-learning platforms on user satisfaction via cognitive and emotional engagement. Mayer (2020) explored the role of emotions on online learning. Zhao and Bacao (2020) analyzed the factors that affect the usage of e-learning, such as social influence, performance expectancy, effort expectancy, facilitating conditions, etc. Similarly, Aldholay et al. (2019) evaluated the mediating role of compatibility and task technology-fit on online learning usage through the Information System Success Model (ISSM). According to a study by Shim and Jo (2020) on the IS success model in the healthcare sector, it was discovered that information quality and service quality significantly predict intention to reuse the site, user satisfaction, and perceived benefits.

Hence, the researchers have explored online learning links with different variables, but there are still a few gaps existing in the literature that need to be explored. Particularly, only few studies have examined the relationship of overall quality factors (information quality, system quality, and service quality) with performance impact in online learning. For instance, a research study by Isaac et al. (2017b) has integrated DMISM and TTF models but has ignored the impact of the overall quality of the system on performance impact. Similarly, an empirical study by Isaac et al. (2017a) has also overlooked the association between overall quality and performance impact. Moreover, researchers have not described comprehensive mechanisms that can affect online learning performance. Likewise, many studies in literature have determined a significant association between the quality of the information system, user satisfaction, and actual usage of the system (Flack, 2016; Aldholay et al., 2018a, 2019). However, there are contradictory findings in various studies on the impact of overall quality on user satisfaction and actual usage (Jung et al., 2015; Tam and Oliveira, 2016; Chiu et al., 2016; Shim and Jo, 2020). The contravention among these studies indicates the likelihood of introducing new intervening variables based on the research perspective and utilization. Likewise, there is a contradiction between studies that examined the relationship between user satisfaction, system usage, and performance impact. Some studies support this relationship (Xinli, 2015; Ashfaq et al., 2020; Shim and Jo, 2020) but (Ellis et al., 2007; Cho et al., 2015) determined that no significant relationship exists between user satisfaction and performance impact. This disagreement provides an opportunity to determine the relevance or irrelevance of the impact of user satisfaction on actual usage and performance. Furthermore, the role of mediators and moderators in online learning models has been merely discussed, such as the role of human-related factors like leadership, cognitive absorption, or perceived usefulness either as mediator or moderator has been rarely discussed. Likewise, system-related factors like compatibility have not been empirically tested.

Thus, few research questions have been formulated based on the challenges faced by the online learning system during COVID-19 and the gaps identified from the previous literature. These are (a) How TTF (i.e., student academic tasks fit with the relevant online learning system) can lead overall quality of the online learning system and user satisfaction to improved performance of students during COVID-19? (b) How can actual usage (i.e., duration and frequency of online learning system usage by the students) lead to the overall quality of the online learning system and user satisfaction to improved performance of students during COVID-19? and (c) Can the perceived usefulness of online learning increase the satisfaction of students concerning the quality of online learning mechanisms?

Hence, in this study, a research framework has been developed based on integrating the TTF model and the DMISM. The TTF model emphasizes the TTF construct and its association with performance impact but does not consider the relationship with the overall quality, user satisfaction, and actual usage constructs. DMISM emphasizes overall quality, user satisfaction, actual usage, and usage performance impact constructs and overlooks the TTF constructs. Hence, six variables were opted to develop an online learning framework based on the literature mentioned above. These variables are overall quality (predictor), user satisfaction, actual usage, TTF, performance impact (outcome), and perceived usefulness as a moderator between overall quality and user satisfaction. Here, overall quality, user satisfaction, actual usage variables have been adopted from DMISM (DeLone and McLean, 1992; Delone and McLean, 2003), and TTF construct has been adopted from TTF model (Goodhue and Thompson, 1995), whereas performance impact variable is common in the two models. Moreover, the moderator “perceived usefulness” has been extracted from TAM (Davis, 1989). The overall quality is a second-order construct comprising information quality, system quality, and service quality. Information quality refers to the extent to which users perceive online learning as organized, relevant, comprehensive, accurate, and up to date (Halonen et al., 2009). System quality is regarded as a degree to which users find a system that is easy to learn, enjoyable, user-friendly, and easy to use (Petter and Mclean, 2009). Similarly, service quality can be defined with empathy, tangible, assurance, reliability, interactivity, responsiveness, and assurance. The variable “user satisfaction” can be defined as how users discern that the system satisfies their needs (Bano et al., 2017). Whereas TTF is defined as the degree to which a system meets requirements or matches the interest of a user (Lin and Wang, 2012). Another research study explains this concept as an intensity to which technology helps users in jobs or coursework (Lu and Yang, 2014). Likewise, actual usage can be defined as the intensity of the user employing the system in terms of duration or frequency (Delone and McLean, 2016). Performance impact refers to the extent to which using a system leads to enhancement of work quality by assisting the users to accomplish their tasks rapidly, empower authority overwork, enhance job conduct, eradicate errors, and improve job efficacy (Norzaidi et al., 2007; Alrajawy et al., 2016). Finally, perceived usefulness is the intensity to which a user finds technology useful to solve his problem and perform a task effectively (Bhattacherjee, 2011).

Thus, the current study has proposed an integrated model to overcome the gaps of TTF and DMISM models by connecting overall quality, user satisfaction, actual usage, TTF, and performance impact. Moreover, the relationship between overall quality and performance impact has been studied in two ways: through the serial mediating role of user satisfaction and actual usage; and through mediating role of user satisfaction and TTF in series with perceived usefulness used as a moderator in the relationship between overall quality and user satisfaction. Hence, this study is valuable because it would provide more comprehensive direction to practitioners about how the performance of students may increase through online learning during the pandemic.

## Literature Review and Hypotheses Development

### Literature Review

After the unpredicted rapid outbreak of COVID-19, there was an immense negative impact on the education sector worldwide, leading to the abrupt adoption of online learning. In these circumstances, online education and the involvement of information technology obtained immense stimulation due to the educational institutions being closed down, instigating many challenges in the path of student learning. It has rendered educators a significant advantage in utilizing and implementing the latest technological solutions on online education to instruct and assess the coursework accomplishment of students. Therefore, considering all the above-stated facts, an extensive literature review was carried out to develop a framework to enhance the academic performance of students.

Different researchers contributed to online learning topics in the recent era. For example, Aldholay et al. (2018c) stated that higher education institutions and governments are trying to commence online learning worldwide. This research indicated that transformational leadership is an essential factor in implementing online learning. Further, this study declared its efforts as the first research to examine transformational leadership with the information system success model. The data collected from the students of nine Yemeni public universities reveal five main results: first, overall quality positively predicts transformational leadership; second, transformational leadership in turn positively influences actual usage; third transformational leadership plays a role of mediating variable for overall quality and actual usage variables; the fourth was that actual usage significantly influences performance impact and user satisfaction; and lastly, user satisfaction previses performance of students. Similarly, Aldholay et al. (2019) demonstrated that e-learning could appreciably increase education, communicative and administrative qualities, encourage learning in flexible places, and use scarce resources through effective time usage. This research has also examined the information system success model in the nine public universities of Yemeni but with compatibility, and divulged that this is the first research examining this relationship. This study revealed that overall quality (having dimensions of information quality, service, and system quality) positively influences compatibility, which, in turn, positively associates with practical usage, TTF, user satisfaction, and performance impact. Moreover, Shodipe and Ohanu (2021) have studied the impact of mobile learning in Nigeria, and data were collected from electronic technology education teachers. This study proposed that (1) the perceived ease of use of teachers positively impacts the actual usage of mobile learning, (2) the disposition of teachers positively influences perceived ease of use, (3) technical training is negatively influenced, and (4) the psychological well-being of teachers is positively influenced by mobile learning. Besides these, Aparicio et al. (2016) referred to their model as important for making e-learning more effective in addition to understanding its success. The results explained that the grit of e-learning positively impacted individual performance and satisfaction.

Furthermore, Ramirez-correa et al. (2016) indicated the four types of e-learning, namely, learning support system, learning design system, learning content system, and learning management system. Data of 258 engineers reveal that information quality positively correlates with the use and user satisfaction of learning management systems. Similarly, system quality has a positive association with user satisfaction and the use of a learning management system. Likewise, the use of a learning management system positively predicts net benefits and user satisfaction of learning management system in the same way that user satisfaction positively impacts net benefits of learning management system. Other than these, Khan et al. (2018) analyzed the technical characteristics and task characteristics of massive open online courses, particularly, anticipated TTF. Moreover, perceived relatedness, perceived competence, and social recognition significantly predict the behavioral intention of students. Further, this study also showed that perceived reputation plays a role as a moderating variable between behavioral intention usage behavior. Similarly, Wu and Chen (2016) analyzed that TAMs and TTF were integrated to know the continuous intention of online open massive courses. Their study revealed that combining the two models helped in understanding the continuous intention in a better way. Moreover, attitude and perceived usefulness are essential for the continuous intention of online open massive courses. Social influence, social recognition, reputation, and TTF perceived ease of use significantly and positively predict continuous intention via mediating role of perceived usefulness. Openness, TTF, and individual technology fit positively affect continuous intention.

Likewise, Tam and Oliveira (2018) showed that the link between TTF and technology use is moderated by individualism. The link between TTF and individual performance is moderated by uncertainty avoidance based on data collected from 204 mobile banking users. Zhao and Bacao (2020) highlighted that confirmation, performance expectancy, social influence, trust, satisfaction, and TTF are the factors that can influence continuous usage while satisfaction is more important among these factors in this regard. For this analysis, data were collected from 532 users of food delivery apps during COVID-19 pandemic. Li and Shang (2020) observed that there is a great challenge in implementing e-government because of the low usage intention of the nation in China. Data from 1650 Chinese nationals revealed that the e-government concept comprises eight contributing dimensions: responsiveness, reliability, system quality, interactivity, accessibility, security, service capability, and information quality. Service quality positively predicts continuous intention *via* perceived service value. Gorla et al. (2010) researched to determine the most important dimension of overall quality. This study analyzed that service quality is the most important variable in influencing organizational performance while system quality is the least variable, and information quality ranked second in this regard.

### Hypotheses Development

#### Overall Quality and User Satisfaction

Numerous studies have analyzed the overall quality of the system comprising information quality, system quality, and service quality (Ho et al., 2010; Isaac et al., 2017b; Pham et al., 2019; Mcknight et al., 2020; Kim et al., 2021). Whereas user satisfaction can be held as the main factor in assessing the success of new technology (Montesdioca and Maçada, 2014). Isaac et al. (2018) argued that overall quality positively predicts user satisfaction. An empirical study by Hossain (2016) observed a positive association between overall quality and user satisfaction. Moreover, various studies have observed a significant impact of overall quality on user satisfaction (Petter et al., 2008; Hassanzadeh et al., 2012; Lin and Wang, 2012; Jung et al., 2015; Oktal et al., 2016; Aparicio et al., 2017; Shahzad et al., 2020). So, based on the studies mentioned above, we infer that overall quality has a significant direct impact on user satisfaction. Therefore, the present study highlights that the greater the overall quality of the online learning system, the more it will lead to the elevated satisfaction level of the students. Therefore, students will rely more on these online systems to accomplish their educational tasks.

Thus, it is proposed the following:

H_1_**:** Overall quality positively predicts user satisfaction.

#### User Satisfaction and Task Technology Fit

Task Technology Fit is regarded as extremely essential when it is concerned with analyzing technology usage in organizations (D’Ambra et al., 2013). Numerous studies have been carried out to examine the relationship between TTF and user satisfaction, and these studies indicate that a significant direct association exists between these two constructs (Lee et al., 2005; Norzaidi et al., 2007; Daud, 2008; Larsen et al., 2009; McGill and Klobas, 2009; D’Ambra et al., 2013; Lee and Lehto, 2013; Glowalla and Sunyaev, 2014). Therefore, in the present study, it is assumed that user satisfaction has a positive impact on TTF considering the fact that the more the students are satisfied with the quality of technology used in online learning, the more the students will consider the technology as highly fit to complete their online academic tasks.

H_2_: User satisfaction positively predicts task technology fit.

#### Task Technology Fit and Performance Impact

With the rapid advancement in technology and the incorporation of various new systems, the main focus is on the technological system usage outcome regarding performance enhancement of users to determine the effectiveness of the system (Shih and Chen, 2013; Montesdioca and Maçada, 2014; Isaac et al., 2017b). In the context of this study, performance impact refers to the extent to which online learning influences student performance in relevance to the preservation of resources, proficiency, capability, and knowledge gain (Isaac et al., 2017a). Many studies in the literature have empirically tested the association between TTF and performance impact and found that TTF positively predicts performance impact (Lee et al., 2005; Norzaidi et al., 2007; Daud, 2008; Larsen et al., 2009; McGill and Klobas, 2009; D’Ambra et al., 2013; Lee and Lehto, 2013; Glowalla and Sunyaev, 2014; Shim and Jo, 2020). Indeed, the performance of students in terms of effectiveness and efficiency increases because of TTF (Sinha et al., 2019).

Hence, it is hypothesized the following:

H_3_: Task technology fit positively predicts performance impact.

#### User Satisfaction and Actual Usage

Another significant dimension in technology-oriented studies is the concept of technology usage by the users. Various studies have been conducted to examine the relationship between user satisfaction and actual usage, and it was observed that user satisfaction has a significant positive association with the actual usage of the system (Petter et al., 2008; Norzaidi et al., 2009; Jafari et al., 2011; Norzaidi and Salwani, 2014). Indeed, the duration of technology usage increases because of user satisfaction (Isaac et al., 2017b). The key to this postulate is also user satisfaction because it is evident that when the user is satisfied, the actual usage of the system will increase. Therefore, it is hypothesized:

H_4_: User satisfaction positively predicts actual usage of the system.

#### Actual Usage and Performance Impact

The association between actual system usage and performance impact is another significant dimension in technology usage (Venkatesh et al., 2003). Some studies have made an effort to reduce the gap by working on the association between actual usage and performance impact (Norzaidi et al., 2007; Hou, 2012; Son et al., 2012). Norzaidi et al. (2009), in their quantitative research, observed that the actual usage of a system has a significant impact on performance. However, studies based on information systems have highlighted that actual usage has a positive impact on performance (Fan and Fang, 2006; Wang and Liao, 2008; Hou, 2012; D’Ambra et al., 2013; Makokha and Ochieng, 2014; Isaac et al., 2016; Wang and Teo, 2020). This relationship signifies that the more frequently the students will use the online system to accomplish their academic tasks, the more it will enhance their performance.

So, it is hypothesized that:

H_5_**:** Actual usage of the system positively predicts performance impact.

#### Mediating Role of User Satisfaction

As the literature indicates, user satisfaction is positively predicted by overall quality (Jung et al., 2015; Isaac et al., 2018), and user satisfaction has a significant positive impact on TTF (Aldholay et al., 2019). It is suggested that overall quality positively influences TTF via user satisfaction. Moreover, as observed from the literature mentioned above, the duration of technological system usage increases because of user satisfaction (Isaac et al., 2017b), and user satisfaction is, in turn, influenced by overall quality (Aparicio et al., 2017; Aldholay et al., 2018b).

So, based on these arguments, we propose the following hypotheses:

H_2a_: Overall quality positively predicts task technology fit through mediating role of user satisfaction.

H_4a_: Overall quality positively predicts actual usage of the system through mediating role of user satisfaction.

#### Mediating Role of Task Technology Fit

As the overall quality of the system is up to the mark, it leads toward user satisfaction and TTF in the sequence. The key to this postulate is user satisfaction and TTF. However, a significant relationship has also been observed between TTF and performance impact (Shim and Jo, 2020). The association between TTF and performance impact was empirically tested, and found that TTF positively predicts performance impact (Shim and Jo, 2020). Indeed, performance of students in terms of effectiveness and efficiency increases because of TTF (Henseler et al., 2015), and this technology fit meets the requirements when the user is more satisfied as specified by Aldholay et al. (2019), resulting from the overall quality.

Hence, it can be concluded the following:

H_3a_: Overall quality positively predicts performance impact through mediating role of user satisfaction and TTF in the sequence.

#### Mediating Role of Actual Usage

It has been observed that a higher level of overall quality of the system leads to the enhanced satisfaction level of the students and indirectly affects actual usage of the system *via* user satisfaction. Here, user satisfaction and actual usage are the keys to this postulate (Isaac et al., 2017b). With this, the literature indicates that performance can be affected because of actual usage (D’Ambra et al., 2013), and actual usage of the system is in turn influenced by user satisfaction (Isaac et al., 2017b). This user satisfaction is significantly determined by the overall quality of the system (Aldholay et al., 2018a). Evidently, overall quality has a positive influence on performance impact via user satisfaction actual usage of the system. Therefore,

H_5a_: Overall quality positively predicts performance impact through mediating role of user satisfaction and actual usage of the system.

#### The Moderating Role of Perceived Usefulness

Further, a research study indicates that a technological user becomes satisfied if he perceives technology useful (Xu and Tina, 2018). By extension to empirical research, a research study by Ashfaq et al. (2020) shows that perceived usefulness positively influences user satisfaction. So, the higher the perceived usefulness, the higher the satisfaction level. In this regard, an example can be coded, and that is if a user asks from a chatbot about shipment, and the chatbot assists him in a way that the user perceives the chatbot as a useful device, he feels satisfied. In contrast, if a chatbot fails to assist the user, the user will rate it as useless or disadvantageous because it leads him to an unsatisfied level. Moreover, if the user perceives the cost of the chatbot is higher than its performance, he feels unsatisfied. Consequently, the user will stop using that particular Chabot. Thus, from this evidence, it can be concluded that perceived usefulness moderates the effect of overall quality on user satisfaction.

H_6_: Perceived Usefulness moderates the relationship between Overall Quality and User Satisfaction.

**FIGURE 1:**
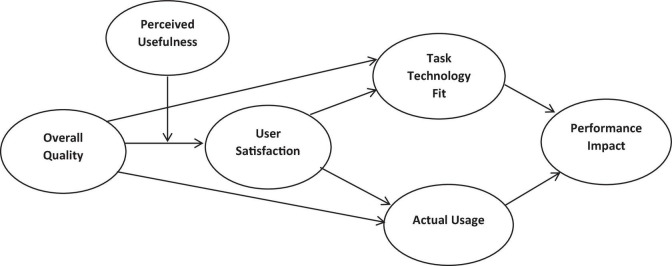
The theoretical framework for the current study.

Based on these hypotheses, a theoretical framework has been developed, as shown in Figure 1.

## Methodology

### Research Design

A multivariate statistical analysis statistical technique, Structural Equation Modeling (SEM), has been utilized to examine the structural relationships and to test the proposed hypotheses empirically, using Analysis of Moment Structures (AMOS^®^) 24 (IBM, Endicott, NY, United States). SEM simultaneously measures the latent variables and performs the path analysis. Moreover, it explicitly assesses measurement error. Model fit measures further enhance its credibility. In order to apply SEM, there are certain conditions to meet. For example, the observed variables should have a multivariate normal distribution, and so the sample size should be more than 200 with no missing value. Moreover, SEM is preferred over Regression Analysis (applied through SPSS) and Partial Least Squares- Structural Equation Modeling (PLS-SEM) (applied through SmartPLS). PLS-SEM does not need to fulfill requirements such as sample size, normality, etc. It does not have well-established model fit measures. Generally, it is not preferred in academia.

Structural Equation Modeling is made up of two constituents: Confirmatory Factor Analysis (CFA) which used to evaluate the measurement model between observed and latent variables, and Path Analysis (PA) which is required to fit the structural model with the latent variables. In CFA, the validity of indicators is tested while the path analysis technique specifies the mode in which a specific latent variable directly or indirectly leads to a change in the other latent variable. This 2-step procedure verifies that the structural model utilizes only the constructs that have a satisfactory measure. Furthermore, three different measures were used to evaluate the fitness of the measurement model and the structural model, namely, relative Chi-square ratio over the degree of freedom (χ^2^/DF), Goodness of Fit Index (GFI), and Root Mean Square Error Approximation (RMSEA).

### Sample and Procedure

The population for this study comprised of Pakistani students of the top 10 public universities and top ten private universities, Higher Education Commission (HEC) ranked in Punjab. The students had an age group ranging from 20 to 40 years. They were enrolled in either bachelor, master, and Ph.D. degrees or any other diploma courses offered by the relevant university. Since the target population did not have a sampling frame, a quota sampling technique was applied to collect the data. The data collection was done by providing a self-administered questionnaire in printed form and by sending the questionnaires to students across universities online *via* email and posting on Facebook pages of the enlisted universities. The total number of questionnaires distributed among the students was 1,500. Of the 1,500 questionnaires, 675 responses were received, while 656 responses were selected for further analysis, discarding 19 responses because of missing or inappropriate information. Thus, the effective response rate of the data collection was 43.73%.

Table 1 indicates the demographics of the respondents, revealing that 65% of the respondents were male and 35% were female. The age statistics show that 15.1% were 20 years of age or less, 74.8% were aged between 21 and 30, and 9.8% were aged between 31 and 40. Moreover, the percent response from the universities ranged between 4.6 and 4.7%. Similarly, 2.4% of respondents were intermediate students, 43.1% were bachelors, 38.7% were associated with master programs, 2% were Ph.D. students, and others were 7%.

**TABLE 1:** The demographics of the respondents.

		Frequency	Percent	Valid percent	Cumulative percent
**Gender**					

	(1) Males	430	65.5	65.8	65.8
	(2) Females	223	34.0	34.2	100.0
	Total	653	99.5	100.0	
Missing	System	3	0.5		
Total	656	100.0			

**Age**					

	(1) 20 or less	99	15.1	15.1	15.1
	(2) 21–30	491	74.8	75.1	90.2
	(3) 31–40	64	9.8	9.8	100.0
	Total	654	99.7	100.0	
Missing	System	2	0.3		
Total	656	100.0			

**University**					

		1	0.2	0.2	0.2
	BZU Bahauddin Zakariya University	30	4.6	4.6	4.7
	COMSATS Institute of Information Technology	30	4.6	4.6	9.3
	FC Forman Christian College and University, Lahore	31	4.7	4.7	14.0
	GCU (Government College University)	33	5.0	5.0	19.1
	GIFT	30	4.6	4.6	23.6
	IIUI International Islamic University Islamabad	30	4.6	4.6	28.2
	IUB The Islamia University of Bahawalpur	30	4.6	4.6	32.8
	LUMS Lahore University of Management Sciences	31	4.7	4.7	37.5
	NUCES National University of Computer and Emerging Sciences	31	4.7	4.7	42.2
	MUST	30	4.6	4.6	46.8
	PMAS ARID UNIVERSITY	30	4.6	4.6	51.4
	PU University of the Punjab	30	4.6	4.6	55.9
	RIPHAH	30	4.6	4.6	60.5
	UCP University of Central Punjab	30	4.6	4.6	65.1
	UET (University of Engineering and Technology), Lahore	67	10.2	10.2	75.3
	UHS (University of Health Science), Lahore	42	6.4	6.4	81.7
	UMT University of Management and Technology, Lahore	31	4.7	4.7	86.4
	UOL (University of Lahore)	30	4.6	4.6	91.0
	UOS (University of Sargodha)	30	4.6	4.6	95.6
	UVAS (University of Veterinary and Animal Sciences), Lahore	29	4.4	4.4	100.0
	Total	656	100.0	100.0	

**Education**					

	(1) Intermediate	16	2.4	2.4	2.4
	(2) Bachelors	283	43.1	43.2	45.6
	(3) Masters	254	38.7	38.8	84.4
	(4) MPhil	67	10.2	10.2	94.7
	(5) Ph.D.	28	4.3	4.3	98.9
	(6) Others	7	1.1	1.1	100.0
	Total	655	99.8	100.0	
Missing	System	1	0.2		
Total	656	100.0			

					

Moreover, common method variance (CMV) was checked, as recommended by Podsakoff et al. (2003). CMV may be present when all the scale items are measured with a single questionnaire, and the entire data are collected at a single point in time. CMV also arises when the association between two constructs is overemphasized. In other words, a methodical covariance is built up instead of the true association between the scale items by CMV. There are several sources of CMV like the complexity of the scale items, the inability of the respondents, double-barreled items, inexperience of the respondents to consider the research topic, low involvement of the respondent in the topic, positioning of the scale items, the temperament of the respondent to give extreme responses, etc. As a result, the modified values of the correlations observed and related indicators can cause erroneous estimates of convergent validity and reliability in the research.

There are two different ways to deal with CMV, namely, procedural remedies, and various statistical techniques. The effective approach to avoid common method variation through procedural remedies is to determine the similarity between the measures of the predictor and criterion variables and then minimize or eradicate them. The researchers used procedural remedies in the initial stages of the questionnaire design to avoid CMV. Common procedural remedies may include using more than one source of information to obtain data for the constructs used in the model. Another way could be to use more clear, compact, and appropriate questions to avoid misapprehension of the scale items by the respondents and minimize the chances of unintended responses. Researchers using procedural remedies may be able to reduce, if not completely remove, the potential impacts of CMV in their research findings. In other instances, they may have problems finding a procedural solution that fulfills all of their requirements. In these circumstances, they might be more inclined to employ one of the statistical treatments applicable. Therefore, a wide range of statistical techniques is made available to the researcher to prevent CMV. The significant ones are Harman’s single-factor test common latent factor (CLF) and CFA of a single factor (Eichhorn, 2014). Harman’s single-factor test, also known as Harman’s one-factor test, is the most popular statistical technique used (Ashfaq et al., 2020). Following this method, all 33 items in this research were put in single exploratory factor analysis, and an unrotated factor analysis was observed, which accounted for only 23.5%. Therefore, the output of the CMV verified that the CMV bias in the sample data did not exist.

### Measurement Scale

Already developed scales were used to collect data for the current study, as displayed in Supplementary Appendix A. All the scale items were evaluated on a seven-point Likert scale (1 Strongly Disagree and 7 Strongly Agree). The scale for Overall Quality was adopted from Aldholay et al. (2019). It has 11 items and a reported value of Cronbach alpha of 0.926. The sample item is “I find the online learning to be easy to use.” User Satisfaction has three items taken from Aldholay et al. (2018a). The alpha value for this scale is 0.915. A Sample item is “My decision to use online learning was a wise one.” Similarly, Task Technology was adopted from Isaac et al. (2017b). It has three items with a reported alpha value of 0.911, and the sample item is “Online learning fits with the way I like to learn and study.” Actual usage with two items was adopted from Aldholay et al. (2019) and the alpha value for this scale is 0.818. The sample item is “on average, how much time do you spend per week using the online learning?” Performance Impact was adopted from Aldholay et al. (2018a). This scale has an alpha value of 0.959 with ten items, and the sample item is “online learning helps me accomplish my tasks more quickly.” Perceived Usefulness was taken from Ashfaq et al. (2020). This scale has four items, with the sample item “using online learning increases my productivity.” It has an alpha value of 0.893. The overall summary of all the items has been reported in Table 2.

**TABLE 2:** Measurement scales and corresponding references for all the constructs.

Construct	Measurement scale	References
Overall quality	11 Items	Aldholay et al., 2019
User satisfaction	3 Items	Aldholay et al., 2018a
Perceived usefulness	4 Items	Ashfaq et al., 2020
Task technology fit	3 Items	Isaac et al., 2017b
Actual usage	2 Items	Aldholay et al., 2019
Performance impact	10 Items	Aldholay et al., 2018a

## Data Analysis and Results

Analysis of a Moment Structure software was applied for analyzing this research. AMOS is a statistical package with an innovative engine for SEM. It has a user-friendly graphical interface, provides a clear research model for learners, quality diagrams for publication, and numeric values are the most reliable.

### Descriptive Analysis

The statistics of descriptive analysis for all variables have been presented in Table 3. The mean value for overall quality was 4.97 with a standard deviation (SD.) of 1.37, while the mean value for user satisfaction was 5.01 and the standard deviation was 1.44. Similarly, the mean obtained for perceived usefulness was 5.01 and a standard deviation of 1.32. TTF had a mean of 4.03 and a standard deviation of 1.96. The mean for Actual Usage observed was 5.49, and the value of standard deviation was 1.52. Lastly, the value of the mean obtained for Performance Impact was 5.04 and a standard deviation of 1.71. It implies that the coefficient of variation (CV = Mean/SD) is not large and the data are not so dispersed, which is an indicator of reliable responses.

**TABLE 3:** Descriptive statistics.

	N	Minimum	Maximum	Mean	*SD*	Skewness	Kurtosis
Perceived usefulness	656	1.25	7.00	5.0130	1.31725	−0.245	–0.892
Overall quality	656	1.00	7.00	4.9753	1.37607	−0.680	–0.020
User satisfaction	656	1.00	7.00	5.0107	1.43767	−0.336	–0.647
Performance impact	656	1.00	7.00	5.0447	1.71182	−0.921	–0.427
Task technology fit	656	1.00	6.67	4.0396	1.95725	−0.090	–1.408
Actual usage	656	1.00	7.00	5.4962	1.51871	−1.100	0.545

The results of the assessment of skewness presented reveal the normal distribution of the data. As kurtosis values are less than 10 and the skewness values were between −1.0 and + 1.0. This indicates appropriate ranges for normality, as shown in Table 3.

### Measurement Model

**FIGURE 2:**
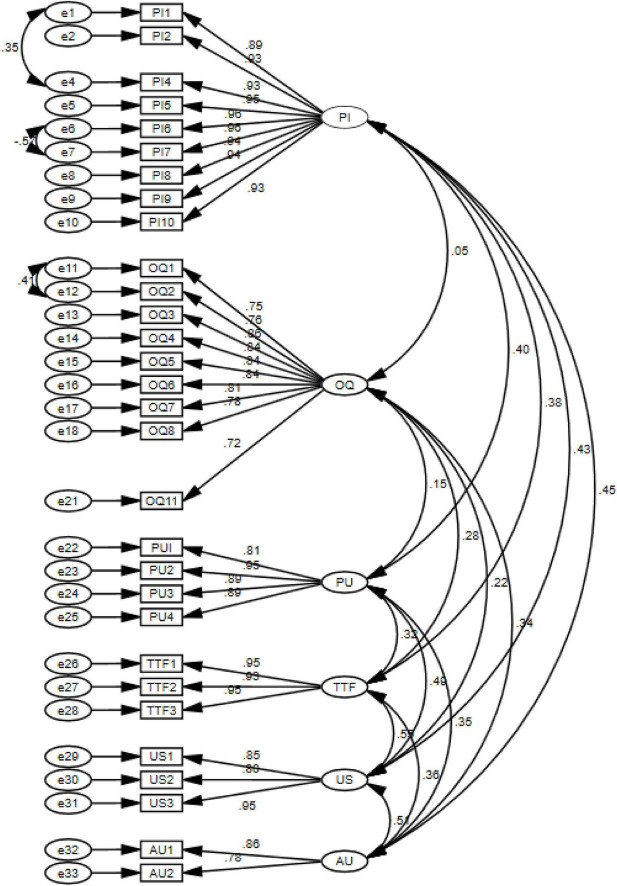
Confirmatory Factor Analysis (CFA) for Measurement Model.

A measurement model depicts the explicit or implicit models that associate the latent variable with its indicators. It is used to assess the goodness of measures of the concepts we are interested in measuring in our theoretical model. CFA is a procedure used to verify a hypothesized measurement model. It represents an association between latent variables or factors (unobserved) and indicators (observed) variables. Therefore, CFA is a statistical procedure proposed for analysis as it has grounds on a theory explaining measurement errors and evaluating the unidimensionality of the model. The assessment of the measurement model through CFA was conducted with the help of construct reliability and validity, as depicted in Figure 2.

Structural Equation Modeling has been utilized in this research as it is a very efficient technique, i.e., there is no other method to provide us with more accurate estimates of those parameters, assuming multivariate normal data. This study utilized the maximum likelihood (ML) estimation method, which affirms that if all the items are mainly loading on their correlated factors, then the uni-dimensionality of the constructs exists, and hence, validity is demonstrated. In Figure 2, the measurement model has been presented, and in Table 4, composite reliability values for the scale reliability and CFA results have been represented.

**TABLE 4:** Discriminant validity.

	CR	AVE	MSV	Max	x(H) P.I.	OQ	PU	TTF	US	AU
PI	0.985	0.878	0.204	0.986	0.937					
OQ	0.941	0.640	0.113	0.945	0.054	0.800				
PU	0.936	0.787	0.237	0.948	0.402^***^	0.152^***^	0.887			
TTF	0.961	0.890	0.300	0.962	0.380^***^	0.275^***^	0.316^***^	0.944		
US	0.903	0.758	0.300	0.932	0.426^***^	0.222^***^	0.486^***^	0.548^***^	0.870	
AU	0.805	0.674	0.256	0.813	0.452^***^	0.337^***^	0.350^***^	0.356^***^	0.506^***^	0.821

***p < 0.001.

*The square roots of Average Variance Extracted (AVE) for the constructs are shown in the diagonal, and inter-construct correlations are shown as off-diagonal elements. PI, Performance Impact; OQ, Overall Quality; PU, Perceived Usefulness; TTF, Task Technology Fit; US, User Satisfaction; AU, Actual Usage.*

#### Model Fit

In research, the word fit is described as the capability of a model to illustrate the data. Precisely, in CFA, a model fit represents how close the observed data complement the relationships prescribed in the hypothesized model. A good-fitting model is agreeably compatible with the data, i.e., to assess if the model significantly fits by the data or does not. Therefore, the goodness-of-fit of the model in relevance to data was evaluated by applying various tests. So based on the goodness-of-fit indices, there is an intimation of the model acceptance.

A chi-square (χ^2^) statistic represents a test that measures how a hypothesized model compares to empirical or observed data. Chi-square is not utilized to measure the model fitness as it depends on the sample size. For this purpose, the minimum discrepancy per degree of freedom (CMIN/DF) ratio is calculated where the ratio of ≤ 2 represents a well-fitted model, an acceptable fit is a ratio of 3–5, and the ratio of ≥ 5 means an unacceptability of the value. In this research the model used for examination has a normed chi-square (χ^2^/DF) = (1017.845/387) = 2.63 (<3.00) which represents a satisfactory fit. The goodness-of-fit index (GFI ≤ 1) calculates the fraction of variance comprised of the estimated population covariance (Tabachnick and Fidell, 2007). It is not correlated to a null hypothesis or baseline. However, it can be generalized by calculating 1-νresidual/νtotal. If the value of GFI > 0.95, it is regarded as a good fit, and if GFI < 0.65, it is regarded as an acceptable fit. The GFI value calculated for the present study model was GFI = 0.911, representing an acceptable model fit. The most important component in covariance structure modeling is root mean square error of approximation (RMSEA). It is considered a good fit if the value of RMSEA is < 0.05. If the value ranges from 0.08 to 0.10, it denotes average fit, and if the value is higher than 0.10, it represents a poor fit. For this study, RMSEA = 0.054 and standardized RMR = 0.0243 which represents justifiable constructs unidimensionality.

#### Reliability of the Variables

In research, reliability refers to the extent to which the research methods provide consistent and stable results. In this study, Cronbach’s alpha was utilized to measure the internal consistency reliability. The Cronbach’s alpha value lies between 0 and 1, and a higher value represents a higher internal consistency. The values of Cronbach’s alpha for the measures listed in Supplementary Appendix B are higher than the 0.70, which is the threshold value that indicates good reliability for the measures used in this research (Fornell and Larcker, 1981).

#### Construct Validity

Validity is defined as the degree to which a test or an instrument measures what it is designed to measure (Thomas et al., 2015). In the present study, construct validity was established after confirming face validity, convergent validity, and discriminant validity. As measurement items have been adopted from the previous studies, it demonstrates face validity. Next, convergent validity was determined, which is the degree to which a measure is positively correlated with alternative measures of the same construct. It was established through indicator reliability and average variance extracted (AVE).

Factor loading was utilized to evaluate indicator reliability. High loadings on a construct depict that the related indicators appear to have much in common represented by the construct (Sarstedt et al., 2020). Factor loadings higher than 0.50 were considered very important (Sarstedt et al., 2020). It was observed that all the items appeared significant (*p* < 0.001), and the loading values were greater than the suggested value of 0.5 (represented in Supplementary Appendix B), which indicates that items in the model have accomplished all the specifications. Moreover, all AVE values were above the threshold value of 0.50 (Sarstedt et al., 2020). The convergent validity has been strongly achieved for all constructs, and satisfactory convergent validity is illustrated in Table 4.

Likewise, discriminant validity is defined as the extent to which items differentiate between constructs or evaluate discrete concepts for the measurement model. It was verified through the Fornell-Larcker criterion and the Heterotrait-Monotrait ratio (HTMT). According to the Fornell-Larcker criterion, the square roots of the AVEs (shown in the diagonal of Table 4) are greater than the inter-construct correlations (values in the respective row and column). This specifies that the constructs are firmly associated with their corresponding indicators than the other constructs present in the model (Chin, 1998; Thomas et al., 2015), representing a good discriminant validity (Sarstedt et al., 2017). However, the correlation among exogenous constructs is determined to be less than 0.85 (Awang, 2000). Therefore, discriminant validity was attained for the constructs present in the model.

However, the Fornell-Larcker criterion has faced some criticism by the researchers. For example, Henseler et al. (2015) specified that it fails to precisely explain the absence of discriminant validity in common research scenarios. Therefore an alternative technique, HTMT of correlations, was suggested based on the multitrait-multimethod matrix. If the value of HTMT determined is greater than 0.90, i.e., HTMT 0.90, or HTMT 0.85, there is a concern for discriminant validity (Henseler et al., 2015). Since all the calculated values were less than the suggested value of 0.85, as shown in Table 5, it specifies the achievement of discriminant validity.

**TABLE 5:** Heterotrait-Monotrait ratio (HTMT) analysis.

	P.I.	OQ	PU	TTF	US	AU
P.I.						
OQ	0.055					
PU	0.401	0.160				
TTF	0.383	0.277	0.318			
US	0.417	0.243	0.502	0.558		
AU	0.448	0.335	0.356	0.364	0.521	

### Structural Model Assessment

In SEM analysis, the second important process is the structural equation model. The structural model can be illustrated after validating the measurement model by describing the relationships between the constructs. Therefore, the structural model elaborates on the association among the variables, exhibiting the particular details of the association between exogenous variables and the corresponding endogenous variables. Evaluating the structural model results helps us determine how closely the theory is supported by empirical data, and enables us to decide if the theory is being confirmed empirically (Hair et al., 2014). The goodness-of-fit for the structural model was corresponding to the goodness-of-fit of the CFA measurement model. In the given structural model, the χ^2^/df = 2.89, CFI = 0.972, and RMSEA = 0.054. These fit indices presented the proof of satisfactory fit among the hypothesized model and the observed data.

### Path Analysis and Hypothesis Testing

**FIGURE 3:**
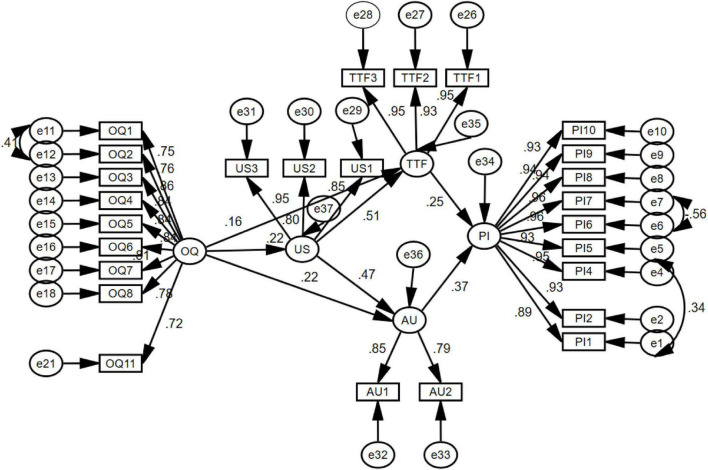
The structural model.

Path analyses were performed to determine the direct and indirect impacts of the exogenous variables. Figure 3 represents a path diagram showing the hypothesized associations among the constructs predicated on findings from previous literature. Overall quality (OQ) is an exogenous variable, while user satisfaction (US), actual usage (AU), TTF, and performance impact (PI) are endogenous variables.

The indirect effects are tested using a bootstrapping technique in structural models by computing the beta (β) values, R^2^, and corresponding *t*-values. Moreover, the existence of the effect is determined by the *p*-value (Sullivan and Feinn, 2012).

As presented in Figure 3 and Table 6, the structural model evaluation stipulates the hypothesis tests. The results of the tests supported the six hypotheses formulated for this study. So it is verified that OQ positively predicts US. Therefore, H_1_ is accepted with β = 0.225, *p* < 0.05. Similarly, US positively predicts TTF, and the results of the tests support this hypothesis. Hence, H_2_ is accepted with β = 0.729, *p* < 0.05 as reported in Table 6. TTF was also observed to directly affect performance. Therefore, H_3_ was also accepted (β = 0.214, *p* < 0.05). Likewise, US positively predicts AU of the system, and test results verify this hypothesis (β = 0.495, *p* < 0.05), as displayed in Table 6. Hence H_4_ is accepted. According to H_5_, AU of the system positively predicts PI, and the results of the tests support it, as exhibited in Table 6. Hence H_5_ is accepted (β = 0.420 and *p* < 0.05).

**TABLE 6:** Evaluation of structural model.

Hypotheses	Relations	Estimate	S.E	C.R	*p*-value	Results
H_1_	US < — OQ	0.225	0.043	5.295	^***^	Accept
H_2_	TTF < — US	0.729	0.054	13.558	^***^	Accept
H_3_	PI < — TTF	0.214	0.032	6.650	^***^	Accept
H_4_	AU < — US	0.495	0.045	10.977	^***^	Accept
H_5_	PI < — AU	0.420	0.050	8.369	^***^	Accept

***p-value < 0.001.

Furthermore, the variance accounted for (VAF) value was used to determine the effectiveness of mediating effects. The value of VAF higher than 80% is regarded as complete mediation, partial mediation ranges from 20 to 80%, and if the value is less than 20%, it depicts no mediation (Hair et al., 2014). The study findings presented in Table 7 show the existence of partial mediation effects in the model. As per H_2a_, OQ positively predicts TTF through the mediating role of US having indirect effects (a × b) (β = 0.164) and (c) direct effects as (β = 0.230), indicating partial mediation, respectively.

**TABLE 7:** Results of mediating effects.

Path	Direct path	Indirect path	Total effect	VAF	Mediation Type
H_2a_ OQ → US → TTF	0.230	0.16	0.394	41.62%	Partial
H_3a_ OQ → US → TTF → PI	–0.006	0.035	0.029	120.69%	Full
H_4a_ OQ → US → AU	0.230	0.111	0.341	32.55%	Partial
H_5a_ OQ → US → AU → PI	–0.018	0.047	0.029	162.06%	Full

To test mediation for H_3a_, similar tests as mentioned above were performed, which showed that OQ positively predicts PI through the complete mediating role of US and TTF in the sequence and (a × b) (β = 0.035) as indirect effects and (c) (β = −0.006) as direct effects, respectively, indicating full mediation.

Again to test H_4a_, similar tests of mediation as mentioned before were performed, and (a × b) (β = 0.111) were indirect effects and (c) (β = 0.230) as direct effects indicating partial mediation. So H_4_ is verified, and hence, OQ positively predicts AU of the system through the partial mediating role of US.

Similarly, to verify H_5a_, similar tests of mediation were performed, and (a × b) (β = 0.047) was determined as indirect effects and (c) (β = −0.018) as direct effects. H_5a_ is verified, therefore, OQ positively predicts PI through the complete mediating role of user satisfaction and actual usage of the system indicating full mediation.

H_6_ states that perceived usefulness acts as a moderator in the association between OQ and US, Hayes Process Macro for moderation was used to test for moderation (Hayes, 2013). At first, the total direct effect of OQ was tested on US. The output represented a significant interaction impact of OQ on US (β = −0.1297; *t* = 3.4633; *p* < 0.001). Likewise, the direct effect of perceived usefulness as a moderator on user satisfaction was tested. The output represented a significant interaction impact of perceived usefulness on US (β = −0.4964; *t* = 13.2931; *p* < 0.001). Finally, we tested the interaction effect of OQ and perceived usefulness on US. The output represented that perceived usefulness has a significant interaction impact on US (β = −0.1005; *t* = 3.5137; *p* < 0.001). Since the interaction term is important, moderating effect therefore occurs in our theory. Therefore, the moderating role of perceived usefulness was found significant in the association between OQ and US, as shown in Table 8. Hence, H_6_ was also verified statistically and approved.

**TABLE 8:** Summarized results of moderating variable.

Variables	Coeff	SE	T	P	LLCI	ULCI
constant	4.9859	0.490	101.7667	0.000	4.8897	5.0821
OQ– > US	1297	0.374	3.4633	0.006	0563	2032
PU– > US	4964	0.373	13.2931	0.000	4231	5697
Interaction						
OQ × PU– > US	1005	0.286	0.005	0.005	0433	1566

## Discussion

This study has developed a model based on the integration between TTF model and information system success model to analyze the association between OQ, US, TTF, AU, perceived usefulness, and PI by collecting data from the top public and private sector universities in Pakistan.

The study established that OQ positively predicts US. It infers that the greater the quality of online learning system in accordance to its ease of use, precision, flexibility, completeness, significance, up-to-date, responsiveness in delivering, usability, and connectivity, the more likely the students acquiring online education would perceive that the service associates more with their needs, perceptions, behavior, and lifestyle. Hence, they will feel more content and satisfied with making a good decision of relying on and acquiring online education. This finding is supported by previous literature (DeLone and McLean, 1992; Delone and McLean, 2003; Aldholay et al., 2018a,b; Chen et al., 2020).

Moreover, it was also revealed that US positively predicts TTF, which implies that US is the critical element in determining the success or failure of the new technology. It is also supported by the findings of a previous study (Isaac et al., 2019). This study also exhibits that OQ positively predicts TTF through the mediating role of US. It implies that the more the students are satisfied with the quality of online education technology, the more they would be content with the services provided by this technology, and the more the students would find this technology fit to fulfill their needs. Hence, this technology would help them more in completing their tasks.

This study also presented that OQ positively predicts PI through the mediating role of US and TTF in the sequence. Based on the empirical test results on the association between TTF and PI, it was found that TTF positively predicts PI, a finding that is identical to the results of other studies (Daud, 2008; Larsen et al., 2009; D’Ambra et al., 2013; Lee and Lehto, 2013; Gatara and Cohen, 2014; Glowalla and Sunyaev, 2014). Moreover, students’ performance in terms of effectiveness and efficiency increases because of TTF, and this technology fit fulfills the requirements when the student is more satisfied, resulting from the OQ.

An association between US and AU was tested and found that US positively predicts AU of the system. This finding is also advocated from the results of previous literature (Hou, 2012; Chen et al., 2020). The research also illustrates that OQ via US does have indirect effects on the AU of technology by the student. In other words, the greater the quality of the online learning system, i.e., the more online learning will be offering usability, flexibility, credibility, responsiveness in acquiring appropriate and up to date information, the students will be satisfied more, and the students will increase the rate and period of their online learning usage more.

This study also supports the hypothesis that AU of the system positively predicts the PI of the students. In literature, few studies have highlighted the association between AU of system and PI like in a quantitative research study by Norzaidi et al. (2009); Hou (2012), Cho and Yu (2015); Cho et al. (2015), and Islam (2016), it was found that actual system usage significantly affects the performance of individuals as users are utilizing the system for task accomplishment, it will lead to their performance enhancement. This study also affirmed that OQ predicts PI through the mediating role of US and AU of the system, which implies that the students will increase their duration of online learning system usage, positively affecting their academic proficiency and coursework productiveness. In this way, this practice adjusts how the students learn things and are considered significant in accomplishing their educational activities.

It is stated that Perceived Usefulness acts as a moderator in association between OQ and US which means that if the students perceive the online learning system is fulfilling their needs and is beneficial for them to accomplish their academic tasks, then the students will be highly satisfied with it. So, the higher the perceived usefulness, the higher the satisfaction level of usefulness. Various studies support the notion that perceived usefulness positively influences US (Al-Rahmi et al., 2015; Omar and Hussein, 2017; Almarri et al., 2020).

## Theoretical and Practical Implications

This study has the potential to make many theoretical contributions. First, this research advances the literature by studying the effect of a moderating variable, perceived usefulness, on the association between OQ and US. Second, this study also extends the literature by examining the sequence mediation mechanism from OQ to PI, mediated by US and TTF in sequence, and testing the effect of OQ to PI, mediated by US and AU. Moreover, this research is essential from a practical point of view due to many reasons. First, the practice of e-learning can appreciably support learning through time usage, and flexible place encourages education using scarce resources and reduces spatial problems. So, this study is more important during the COVID-19 pandemic because educational institutions have focused on remote learning.

Second, this study provides a comprehensive framework to policymakers about how the performance of students can be enhanced by using online learning as educational institutions and governments of almost all countries are trying to place online education at a massive level to ensure effective student learning. As per the findings of the proposed framework, the performance of students can be enhanced in online learning if the OQ, US constructs, TTF, and AU of the system is managed appropriately. Third, the expected results of this study will help the students enhance knowledge acquisition, improve their educational performance, enhance knowledge acquisition, and improve student creativity and innovative skills, which may reduce their stress level in acquiring online education in COVID-19.

### Managerial Implications

The findings of the study would help university policymakers focus on enhancing the awareness and understanding of instructors and students of the e-learning system by organizing training programs about how to use it. The required technical resources to maintain the e-learning system should be available every time. The management must ensure that the online learning system developed is user-friendly, simple, and easy to use. Moreover, the university administration must provide the required hardware, software, and internet access. Instructors and students will be able to effectively utilize online learning if the essential technological resources are updated regularly.

Furthermore, the framework developed in this study would facilitate students, tutors, and other administrative staff to use the new technologies in resolving their multiple problems. Many governments worldwide have adequately increased educational attainment in their country by providing students with advanced technological devices. Pakistan can also effectively make use of this approach. Although Pakistan is a developing country with limited available resources, it can still make full use of the advantages provided by online education, and provide high-quality education nationwide.

## Conclusion

The WHO, earlier in the year 2020, declared the pandemic of the novel SARS-CoV2 viral infection, and it has become the greatest public health challenge globally. The social distancing policy has been implemented in all the countries throughout the world to control the pandemic, leading to the closure of educational institutions in most countries. Educational institutions were constrained to make significant and timely amendments in their present educational system to keep providing education and maintain student academic progression. As a result, the learning and teaching activities were instantly transferred to complete e-learning. A similar strategy was also implemented in Pakistan to control the pandemic. This abrupt shift from face-to-face to online education has imposed a sort of stress to a certain extent on the students and directly affected the academic performance of the students. The present research described the online learning perspectives of students and determined the factors that can enhance the academic performance of students by utilizing the best-fit technology. In order to address this issue, the current research proposed a unified model between the information system success model and the task technology theoretical model. The important constructs of the framework were: OQ, TTF, US, perceived usefulness, AU of the system, and PI.

The results of different tests suggested that the framework proposed was beneficial in exhibiting the impact on the academic performance of students *via* online learning. Moreover, US is significantly essential in assessing TTF and AU of online learning and facilitating the association between OQ, US, and AU, and US, AU, and PI, respectively. TTF also plays a vital role in determining academic performance and its facilitation role among US and academic performance. Perceived usefulness also plays a significant role in determining US. The results of the tests performed highly supported the associations between the constructs of the framework. The findings are consistent with the previous research work on the topic (Norzaidi et al., 2009; Hou, 2012; Cho et al., 2015; Islam, 2016). Educational experts and policymakers should emphasize these features for enhancing the probability of improved performance. Finally, the findings of this research would provide practical support to any initiative of the government of Pakistan associated with the higher education sector to establish circumstances that fit with student tasks, social values, and lifestyles, in which the students are more destined to make use of online learning to improve their academic performance and eventually their work-life quality.

## Limitations and Future Research Directions

There are few limitations of the current study that can provide direction for future studies. Since data have been collected from universities located in Punjab, it is recommended that future researchers collect data from universities of all provinces of Pakistan to generalize the finding of the current study. Future studies can be conducted on a broader scale by comparing the online education system implemented in the universities of Pakistan with online education systems in universities of other countries. Further, this study is limited to collecting cross-sectional data, while future researchers should consider longitudinal data, and conduct experiments to understand causality. Moreover, perceived usefulness has been used as a moderator in the current research. Therefore, future researchers should also consider other moderators. In this regard, ease of use can be used as a moderator, and details related to this moderation can be seen (Ashfaq et al., 2020). Further, the human factor is essential in motivating the students for online earning, suggesting that transformational leadership can also be used as a moderator (Isaac et al., 2018).

Moreover, paths of the framework of the current study can be speculated to develop another scenario. For example, AU and US can be replaced in future studies. Besides, this study is limited to the education sector, while future researchers can consider its application to other sectors in order to corroborate the framework of the current study. Similarly, in this study, student satisfaction and PI are observed to be influenced by the OQ of the online learning system, but other aspects may have considerable effects on both. So, future studies should focus on the impact of different factors such as institutional factors, learner characteristics, the role of instructors, and course content design. Likewise, the online education system has been assessed from the perspectives of students, whereas in future research work, the views of the administrative and academic staff of the institution should also be considered.

## Data Availability Statement

The raw data supporting the conclusions of this article will be made available by the authors, without undue reservation.

## Ethics Statement

The studies involving human participants were reviewed and approved by the Ethical Review Committee of Namal Institute Mianwali, Pakistan, Ref: NML-ERC/2020-032. The patients/participants provided their written informed consent to participate in this study.

## Author Contributions

SB, AM, and SS contributed to the conception and design of the study. TR organized the database. AI performed the statistical analysis. SB wrote the first draft of the manuscript. AM, SS, TR, and AI wrote sections of the manuscript. All authors contributed to manuscript revision, read, and approved the submitted version.

## Conflict of Interest

The authors declare that the research was conducted in the absence of any commercial or financial relationships that could be construed as a potential conflict of interest.

## Publisher’s Note

All claims expressed in this article are solely those of the authors and do not necessarily represent those of their affiliated organizations, or those of the publisher, the editors and the reviewers. Any product that may be evaluated in this article, or claim that may be made by its manufacturer, is not guaranteed or endorsed by the publisher.
