# Supplementary material for: Students’ Performance in Online Learning Environment: The Role of Task Technology Fit and Actual Usage of System During COVID-19
Source: Front Psychol. 2021 Nov 4;12:759227. doi: 10.3389/fpsyg.2021.759227 (PMC8599586; doi:10.3389/fpsyg.2021.759227)
Supplement: Supplementary file 1 [file Data_Sheet_1.docx]

**Appendix A**

***QUESTIONNAIRE***

*Dear Respondent!*

*This study is being conducted by the Institute of Quality & Technology Management, University of the Punjab, Lahore. It aims to investigate “****EXAMINING THE EFFECT OF OVERALL QUALITY ON STUDENTS’ PERFORMANCE: THE ROLE OF TASK TECHNOLOGY FIT AND ACTUAL USAGE OF SYSTEM IN ONLINE LEARNING PRACTICE****” Your participation will be much appreciated and all data will be kept strictly confidential. During the questionnaire, if you feel offended in any manner, I sincerely apologize for the inconvenience of the matter caused.*

| **Gender** | Male | | | | Female | | | |
| --- | --- | --- | --- | --- | --- | --- | --- | --- |
| **Age** | 20 or less | | | 21-30 | | | 31-40 | |
| **University Name** |  | | | | | | | |
| **Education Level** | Intermediate | Bachelors | Masters | | M.Phil. | Ph.D. | | Others |
| **Email id** |  | | | | | | | |

**Record your responses on a scale given below:**

| **Strongly Disagree** | **Disagree** | **Somewhat Disagree** | **Neither Agree nor Disagree** | **Somewhat Agree** | **Agree** | **Strongly Agree** |
| --- | --- | --- | --- | --- | --- | --- |
| **1** | **2** | **3** | **4** | **5** | **6** | **7** |

| **OVERALL QUALITY** | | | | | | | | | | | | | | | | | | |
| --- | --- | --- | --- | --- | --- | --- | --- | --- | --- | --- | --- | --- | --- | --- | --- | --- | --- | --- |
| 1 | | | | I find online learning to be easy to use. | | | | | | 1 | 2 | | 3 | 4 | 5 | 6 | 7 |  |
| 2 | | | | I find online learning to be flexible to interact with. | | | | | | 1 | 2 | | 3 | 4 | 5 | 6 | 7 |  |
| 3 | | | | My interaction with online learning is clear & understandable. | | | | | | 1 | 2 | | 3 | 4 | 5 | 6 | 7 |  |
| 4 | | | | Online learning provides up-to-date knowledge. | | | | | | 1 | 2 | | 3 | 4 | 5 | 6 | 7 |  |
| 5 | | | | Online learning provides accurate knowledge. | | | | | | 1 | 2 | | 3 | 4 | 5 | 6 | 7 |  |
| 6 | | | | Online learning provides relevant knowledge. | | | | | | 1 | 2 | | 3 | 4 | 5 | 6 | 7 |  |
| 7 | | | | Online learning provides comprehensive knowledge. | | | | | | 1 | 2 | | 3 | 4 | 5 | 6 | 7 |  |
| 8 | | | | Online learning provides organized knowledge. | | | | | | 1 | 2 | | 3 | 4 | 5 | 6 | 7 |  |
| 9 | | | | I could use the online learning services at any time, anywhere I want. | | | | | | 1 | 2 | | 3 | 4 | 5 | 6 | 7 |  |
| 10 | | | | Online learning offers multimedia (audio, video, and text) types of course content. | | | | | | 1 | 2 | | 3 | 4 | 5 | 6 | 7 |  |
| 11 | | | | Online learning enables interactive communication. | | | | | | 1 | 2 | | 3 | 4 | 5 | 6 | 7 |  |
|  | | | | **USER SATISFACTION** | | | | | | | | | | | | | | |
| 12 | | | | My decision to use online learning was a wise one. | | | | | | 1 | 2 | | 3 | 4 | 5 | 6 | 7 |  |
| 13 | | | | The online learning has met my expectations. | | | | | | 1 | 2 | | 3 | 4 | 5 | 6 | 7 |  |
| 14 | | | | Overall, I am satisfied with online learning. | | | | | | 1 | 2 | | 3 | 4 | 5 | 6 | 7 |  |
| **TASK TECHNOLOGY FIT** | | | | | | | | | | | | | | | | | | |
| 15 | | | Online learning fits with the way I like to learn and study. | | | | | | | 1 | 2 | | 3 | 4 | 5 | 6 | 7 |  |
| 16 | | | Online learning is suitable for helping me complete my academic assignments. | | | | | | | 1 | 2 | | 3 | 4 | 5 | 6 | 7 |  |
| 17 | | | Online learning is necessary for my academic tasks. | | | | | | | 1 | 2 | | 3 | 4 | 5 | 6 | 7 |  |
|  | | | **PERFORMANCE IMPACT** | | | | | | | | | | | | | | | |
| 18 | | | Online learning helps me to accomplish my tasks more quickly | | | | | | | 1 | 2 | | 3 | 4 | 5 | 6 | 7 |  |
| 19 | | | Online learning makes it easier to complete my tasks. | | | | | | | 1 | 2 | | 3 | 4 | 5 | 6 | 7 |  |
| 20 | | | Online learning saves me money. | | | | | | | 1 | 2 | | 3 | 4 | 5 | 6 | 7 |  |
| 21 | | | Online learning improves my learning performance. | | | | | | | 1 | 2 | | 3 | 4 | 5 | 6 | 7 |  |
| 22 | Online learning enhances my academic effectiveness. | | | | | | | | | 1 | 2 | | 3 | 4 | 5 | 6 | 7 |  |
| 23 | Online learning helps reviews and eliminate errors in my work tasks. | | | | | | | | | 1 | 2 | | 3 | 4 | 5 | 6 | 7 |  |
| 24 | Online learning helps me to realize my future target. | | | | | | | | | 1 | 2 | | 3 | 4 | 5 | 6 | 7 |  |
| 25 | Online learning helps me acquire new knowledge. | | | | | | | | | 1 | 2 | | 3 | 4 | 5 | 6 | 7 |  |
| 26 | Online learning helps me acquire new skills. | | | | | | | | | 1 | 2 | | 3 | 4 | 5 | 6 | 7 |  |
| 27 | Online learning helps me to come up with innovative ideas. | | | | | | | | | 1 | 2 | | 3 | 4 | 5 | 6 | 7 |  |
|  | **PERCEIVED USEFULNESS** | | | | | | | | | | | | | | | | | |
| 28 | Using online learning helps me to accomplish things more quickly | | | | | | | | | 1 | 2 | | 3 | 4 | 5 | 6 | 7 |  |
| 29 | I find online learning useful in my daily life. | | | | | | | | | 1 | 2 | | 3 | 4 | 5 | 6 | 7 |  |
| 30 | Using the online learning increases my productivity | | | | | | | | | 1 | 2 | | 3 | 4 | 5 | 6 | 7 |  |
| 31 | Using online learning helps me to perform many things more conveniently. | | | | | | | | | 1 | 2 | | 3 | 4 | 5 | 6 | 7 |  |
|  | | | | **ACTUAL USAGE** | | | | | | | | | | | | | | |
| 32 | | | | On average, how frequently do you use online learning? | | | | | | | | | | | | | | |
| 1. Certainly not | | | | | 2. Less than once a month | 3. Once a month | 4. A few times a month | 5. A few times a week | 6. About once a day | | | 7. Several Times a day | | | | | | |
| 33 | | Average, how much time do you spend per week using online learning? | | | | | | | | | | | | | | | | |
| 1.Certainly not | | | | | 2. Almost never | 3. Less than two hours | 4. Two to four hours | 5. Four to six hours | 6.Six to eight hours | | | 7.More than eight hours | | | | | | |

**Appendix B**

**Standardized Factor Loadings and Cronbach Alpha Values**

|  | |  | | Factors | | Standardized Loading | Cronbach Alpha |
| --- | --- | --- | --- | --- | --- | --- | --- |
|  | |  | | Performance Impact | |  | .905 |
| PI1 | | <--- | | PI | | .888 |  |
| PI2 | | <--- | | PI | | .930 |  |
| PI4 | | <--- | | PI | | .933 |  |
| PI5 | | <--- | | PI | | .948 |  |
| PI6 | | <--- | | PI | | .963 |  |
| PI7 | | <--- | | PI | | .958 |  |
| PI8 | | <--- | | PI | | .943 |  |
| PI9 | | <--- | | PI | | .941 |  |
| PI10 | | <--- | | PI | | .929 |  |
|  | | Overall Quality | | | |  | .941 |
| OQ1 | | <--- | | OQ | | .748 |  |
| OQ2 | | <--- | | OQ | | .756 |  |
| OQ3 | | <--- | | OQ | | .862 |  |
| OQ4 | | <--- | | OQ | | .840 |  |
| OQ5 | | <--- | | OQ | | .837 |  |
| OQ6 | | <--- | | OQ | | .835 |  |
| OQ7 | | <--- | | OQ | | .812 |  |
| OQ8 | | <--- | | OQ | | .781 |  |
| OQ11 <--- | | | | OQ | .845 |  |  |
|  | |  | | Perceived Useful |  | .936 |  |
| PUI | | <--- | | PU | .812 |  |  |
| PU2 | | <--- | | PU | .946 |  |  |
| PU3 | | <--- | | PU | .893 |  |  |
| PU4 | | <--- | | PU | .892 |  |  |
|  | |  | | Task Technology Fit |  | .960 |  |
| TTF1 | | <--- | | TTF | .951 |  |  |
| TTF2 | | <--- | | TTF | .928 |  |  |
| TTF3 | | <--- | | TTF | .952 |  |  |
|  | |  | | User Satisfaction |  | .900 |  |
| US1 | | <--- | | US | .854 |  |  |
| US2 | | <--- | | US | .801 |  |  |
| US3 | | <--- | | US | .950 |  |  |
|  | |  | | Actual Usage |  |  |  |
| AU1 | | <--- | | AU | .858 |  |  |
| AU2 | | <--- | | AU | .782 |  |  |
